# Predicting Psychiatric Hospitalizations among Elderly Veterans with a History of Mental Health Disease

**DOI:** 10.5334/egems.207

**Published:** 2018-05-17

**Authors:** Zachary Burningham, Jianwei Leng, Celena B. Peters, Tina Huynh, Ahmad Halwani, Randall Rupper, Bret Hicken, Brian C. Sauer

**Affiliations:** 1 University of Utah, US; 2 Salt Lake City VA Medical Center, US

**Keywords:** Health Services Research, Quality Improvement, Decision Support Techniques, Risk Factors, Mental Health, Veterans

## Abstract

**Introduction:**

Patient Aligned Care Team (PACT) care managers are tasked with identifying aging Veterans with psychiatric disease in attempt to prevent psychiatric crises. However, few resources exist that use real-time information on patient risk to prioritize coordinating appropriate care amongst a complex aging population.

**Objective:**

To develop and validate a model to predict psychiatric hospital admission, during a 90-day risk window, in Veterans ages 65 or older with a history of mental health disease.

**Methods:**

This study applied a cohort design to historical data available in the Veterans Affairs (VA) Corporate Data Warehouse (CDW). The Least Absolute Shrinkage and Selection Operator (LASSO) regularization regression technique was used for model development and variable selection. Individual predicted probabilities were estimated using logistic regression. A split-sample approach was used in performing external validation of the fitted model. The concordance statistic (C-statistic) was calculated to assess model performance.

**Results:**

Prior to modeling, 61 potential candidate predictors were identified and 27 variables remained after applying the LASSO method. The final model’s predictive accuracy is represented by a C-statistic of 0.903. The model’s predictive accuracy during external validation is represented by a C-statistic of 0.935. Having a previous psychiatric hospitalization, psychosis, bipolar disorder, and the number of mental-health related social work encounters were strong predictors of a geriatric psychiatric hospitalization.

**Conclusion:**

This predictive model is capable of quantifying the risk of a geriatric psychiatric hospitalization with acceptable performance and allows for the development of interventions that could potentially reduce such risk.

## Introduction

Inpatient utilization commonly increases among aging populations [1]. Aging Veterans are especially vulnerable to institutionalization because they often suffer from both chronic physical and psychiatric conditions, and reportedly experience greater disease burden in comparison to other elderly populations [2,3].

The prevalence of psychiatric hospitalizations among Veterans increases for those with multiple comorbid mental conditions [4]. Older Veterans with multiple comorbid mental conditions and chronic physical conditions are increasingly difficult to treat due to the complexity of multiple disease states [5]. To further complicate matters, physical comorbidities are often under recognized and inadequately treated among those struggling with psychiatric disorders [6]. Special attention is often focused on the underlying psychiatric disorder and little emphasis is placed on ensuring the patient is undergoing frequent examination and receiving coordinated care. Thus, aging Veterans with psychiatric disease are likely some of the most time-consuming and complex patients a provider must manage.

Unfortunately, not all Veterans Affairs (VA) medical centers (VAMCs) have available geriatric care in their inpatient psychiatric units, increasing the likelihood for older Veterans to be admitted to community psychiatric hospitals during acute mental health episodes. These hospitalizations can require substantial financial resources. Additionally, after discharge, patients with severe mental illness are prone to psychiatric hospital readmission when they are not successfully connected with coordinated outpatient services [7]. Thus, due to the complex health status of many aging Veterans, ensuring they are connected to outpatient services and receiving early treatment is vital in reducing costly psychiatric inpatient admissions.

Aging Veterans with psychiatric conditions are often cared for by Patient Aligned Care Teams (PACT), and PACT care managers are tasked with identifying aging Veterans with psychiatric disease who may benefit from better coordinated outpatient care. However, few resources exist to support psychiatric risk assessment of a PACT provider’s patient panel. In order to prevent psychiatric crisis, reduce psychiatric institutionalization, and decrease the burden that PACT care managers and other clinical teams may have in coordinating appropriate care amongst a complex aging population, we have developed and validated a prediction model that identifies Veteran geriatric patients, with existing mental health conditions, who are at risk of experiencing a geriatric psychiatric hospital admission within 90 days.

## Methods

### Study Design and Data Source

This study applied a cohort design to historical data available in the VA Corporate Data Warehouse (CDW). The CDW is a national repository comprising data from several Veterans Health Administration (VHA) clinical and administrative systems [8]. Over 200 Veterans Health Information Systems and Technology Architecture (VistA) modules are responsible for generating CDW data. At point of care, data are entered into VistA systems by way of manual entry, barcode scanning, or through automated instrumentation. These data are then uploaded into the CDW and organized into relational format by logical domains. The CDW production server that houses the data utilized in this study is refreshed daily. Data domains utilized in this study included: patient, inpatient, outpatient, purchased care, and pharmacy.

Access to the CDW for operational purposes is granted by the National Data Systems (NDS) group, a division under the VHA Office of Informatics and Information Governance (OIIG) Health Information Governance (HIG). A formal Institutional Review Board (IRB) was not required as this operational activity was characterized as quality assessment and improvement, which enables the data user broader access to the CDW, while remaining compliant with the Health Insurance Portability and Accountability Act (HIPAA).

### Study Population

This cohort study is comprised of Veterans, 65 years of age or older, with a history of mental health disease; our target population at risk for psychiatric institutionalization. As previously described, when Veterans age, the likelihood of developing chronic physical and psychiatric disease increases. When these conditions occur simultaneously or in combination with other physical ailments they often lead to increased health care utilization, need for intensive caregiving, and costly hospital institutionalization. From a clinical perspective, aging Veterans with these characteristics are difficult to monitor and treat due to their complex disease state and lack of resources that support risk assessment.

All Veterans who were reportedly 65 years of age or older at any point between January 1, 2012, and January 31, 2015, were identified in the CDW. This total population was then randomly split in half, resulting in two separate population datasets. One dataset was used in model development and the other was set aside for external validation. Amongst each population dataset, Veterans with evidence of two or more mental-health related diagnoses or at least one mental-health related medication fill that reportedly occurred anytime between January 1, 2012, and January 31, 2015, were classified as the population at risk that would be analyzed. Healthcare Cost and Utilization Project (HCUP) Single-level Clinical Classifications Software (CCS) categories were used to represent a mental health-related diagnosis, while VA drug class codes were used to represent a mental health-related drug fill. The chosen HCUP CCS single-level diagnosis categories and VA drug class codes used in identifying the population at risk are summarized in Tables A1 and A2 in Appendix A.

Prior to selecting an index date, the eligibility window for each member of the population at risk was determined. The eligibility window is defined as the time span within the study period that the Veteran reportedly was actively using the VA health care system. System use, defined as any outpatient or inpatient event, or drug fill, was summarized for every Veteran in both the model development population dataset and external validation population dataset. The earliest date of system use, within the study period, signified the start date of the Veteran’s eligibility window. Subsequently, the last known date of system use, within the study period, signified the end date of the Veteran’s eligibility window. The index date was randomly chosen from the dates of system use within a Veteran’s eligibility window. However, the randomly chosen index date must have occurred at least 365-days after the start of the eligibility window to ensure a sufficient one-year baseline period was available for analysis. The one-year baseline period is the 365-day observational window prior to the index date, which is the time we allowed potential candidate predictors to be measured, accrue, and contribute to the prediction of a psychiatric hospitalization. Furthermore, a 90-day follow-up window was required for outcome measurement. Therefore, the randomly chosen index date was also required to have occurred at least 90-days prior to the end of a Veteran’s eligibility window.

### Outcome

The outcome included both VA and Non-VA psychiatric hospitalizations. Clinical expert experiences combined with a series of case hospitalization reviews, using the VA’s Computerized Patient Record System (CPRS), helped us identify the most appropriate approach for detecting psychiatric hospitalizations. VA psychiatric hospitalizations were classified as any inpatient stay where the initial or any subsequent transfer treating specialty was “psychiatry.” Non-VA psychiatric hospitalizations were classified as any inpatient stay where the principal diagnosis was one of the mental-health related HCUP CCS single-level diagnosis categories originally used in identifying the population at risk (see Table A1 in Appendix A).

### Predictors

Potential predictors for model development were chosen based on literature review and consultation with clinical experts specializing in geriatric and psychiatric care. Candidate predictor types were categorized as: demographic predictors, health care utilization predictors, clinical condition and laboratory predictors, and calculated pharmacy and health status predictors.

#### Demographic Predictors

Demographic candidate predictors chosen included: age, gender, race, rurality, marital status, homelessness, and whether the Veteran was living with next of kin. Additionally, service connectedness, evidence of private insurance, and means test results, which identified whether the Veteran was required to pay copays for care, were also included as socioeconomic factors.

#### Health Care Utilization Predictors

Health care utilization variables were selected for inclusion in the model because they often occur during or are ordered as part of a mental health or behavioral evaluation and their presence in the medical chart may indicate an escalating behavioral or psychiatric situation that could result in a psychiatric hospitalization. VHA stop codes were used in defining outpatient visit types of interest and they have been summarized in Table A3 in Appendix A. We included the number of urgent care and emergency room encounters that occurred one-month, three-months, and one-year prior to the index date. Furthermore, the number of primary care visits, home-based primary care visits, home health encounters, and mental health-related and non-mental health-related social work encounters were calculated and included as potential predictors during model development. Additionally, evidence of whether the Veteran was ever transferred to a nursing home facility after an inpatient stay was captured and included as a potential predictor. Lastly, utilizing the definition of the outcome as previously described, an indicator identifying whether the Veteran had a previous psychiatric hospitalization during the 365-day baseline period was included during modeling.

#### Clinical Condition and Laboratory Predictors

Several conditions and laboratory procedures that are believed to be associated with the risk of a psychiatric hospitalization were included as potential candidate predictors. We calculated the number of unique mental health-related diagnoses reported for each Veteran during the one-year baseline window using the same HCUP CCS categories used in identifying the population at risk (Table A1 in Appendix A). This candidate predictor was calculated using inpatient, outpatient, and purchased care CDW data domains. Select conditions of interest identified using HCUP CCS single-level diagnosis categories and International Classification of Diseases ninth (ICD-9) and tenth (ICD-10) revision codes included: human immunodeficiency virus (HIV) (CCS: 5), alcohol-related disorders (CCS: 660), psychosis (CCS: 659), bi-polar disorder (ICD-9: 296.4–296.8; ICD-10: F31.0–F31.9), delirium (ICD-9: 291–293, 780.09; ICD-10: F10.231, F11.23, F13.231, F19.231, F05, R41.0), history of attempted suicide and intentional self-inflicted injury (CCS: 662), history of dementia and other cognitive disorders (CCS: 653), and traumatic brain injury (TBI) (CCS: 85).

Select laboratory procedures were identified by Current Procedural Terminology (CPT) codes and evidence of whether they occurred or not during baseline was extracted. The following lab tests were identified as potential candidate predictors: valproic acid (VPA) (CPT: 80164), rapid plasma reagin (RPR) (CPT: 86592–86593), vitamin B-12 (CPT: 82607), blood alcohol screening (CPT: 80321–80322), barbiturate screening (CPT: 80345), opioid screening (CPT: 80361–80365), lead screening (CPT: 83655), benzodiazepine screening (CPT: 80346–80347), amphetamine screening (CPT: 80324–80326), cannabis screening (CPT: 80349–80352), and cocaine screening (CPT: 80353). Additionally, an indicator that identified whether a brain scan was performed via CT or MRI was also included using CPT codes (CPT: 70460, 70450, 70470, 70551, 70553).

#### Calculated Pharmacy and Health Status Predictors

The proportion of days covered (PDC) was calculated during baseline for all mental health-related drug fills combined (see Table A2 in Appendix A). The mental health-related drug fills were extracted using the same VA drug class codes used in identifying the population at risk. The PDC is a common approach to measuring medication adherence based on pharmacy refill data and subsequently was used to represent the utilization of mental health treatment during baseline. Further, we also classified all medications filled during baseline as appropriate or potentially inappropriate, based on the 2015 American Geriatrics Society (AGS) Beers Criteria [9]. This information was used to calculate a continuous candidate predictor that represented the number of potentially inappropriate medication (PIM) fills that accrued during the baseline period. A binary response predictor was also created and included during model development that identified members of the study population that never filled a medication during baseline that was potentially inappropriate.

We also utilized the VHA Care Assessment Needs (CAN) scores that are designed to reflect a patient’s likelihood of death and/or hospitalization in the next 90 days and 1-year, expressed as probabilities and risk percentiles [10]. The CAN score risk percentile indicates how a patient’s likelihood of death and/or hospitalization compares with others across the VA and is modeled using patient demographics, evidence of coexisting conditions, vital signs (blood pressure, heart rate, respiratory rate, body mass index), health care utilization (inpatient and outpatient), and number of fills for certain classes of medications such as: opioids, anti-depressants, benzodiazepines, beta-blockers, and ACE inhibitors.

### Statistical Analysis

The Least Absolute Shrinkage and Selection Operator (LASSO) regularization regression technique, commonly used in developing prediction models on high-dimensional data, was the variable selection technique utilized for model development [11]. Unlike traditional variable selection techniques, the LASSO is effective in reducing overfitting by penalizing regression coefficients for being too large, thus reducing the likelihood for extreme predictions. Candidate predictors that do not contribute to the model sufficiently will have their coefficients essentially shrunk to zero. Therefore, the LASSO can be viewed as having a built-in variable selection technique.

Logistic regression was the model estimation approach used in examining the relationship between our dependent variable and independent predictors. The estimated regression coefficients produced by our analysis are “adjusted,” meaning the individual coefficient for a given predictor is conditional on the values of the other predictors included in the model. In order to quantify model performance during model development, key discrimination measures were calculated, including the concordance statistic (C-statistic) and subsequent area under the Receiving Operator Characteristic (ROC) curve, which is a plot of sensitivity against 1-specificity.

During model fitting, the LASSO was executed on the model development population with 10-fold cross-validation enabled, which essentially splits the data randomly into 10 folds of equal size. All folds but one are used for model training, with the remaining fold being used for assessing model performance. This process is repeated 10 times, in which all of the individual folds have had a chance to serve as a test set. Once this process is completed a series of Models, *M_0_, M_1_* … *M_k_*, are identified with each model being the solution for a unique tuning parameter value. Cross-validation estimates the expected generalization error for each tuning parameter value or lambda, which controls the strength of the LASSO penalty. *M_0_* would be considered the model that would impose the maximum lambda amount on the regression coefficients, resulting in the least complex model possible. *M_k_* would be considered the most complex model, for which no penalty is imposed. We initially examined the model utilizing the lambda that minimized the cross-validated mean square error (CVMSE). Typically, it is recommended to use the lambda that minimizes the CVMSE plus one standard error (SE) when selecting the best model [12]. Such an approach has been the standard as this results in the final model being less complex without drastically reducing accuracy. For our final model, we chose to use the lambda that minimizes the CVMSE plus 0.5 SE, in order to strike a balance between model complexity and superior accuracy.

### External Validation

A split sample approach was utilized in performing external validation of the fitted model. As previously described, the study population was randomly split in half prior to modeling, with the second study population dataset being set aside to validate model performance. To further reiterate, the external validation population was required to meet all the same inclusion criteria as the original population sample used in training the model. Furthermore, the window of eligibility and subsequent index date for each member of the validation population was identified using the same approach as previously described for the model development population. The C-statistic and area under the ROC curve were again utilized to quantify the performance of the model previously fitted on the model development population.

## Results

### Population

We identified 8,050,371 Veterans that were 65+ years of age during the study period, from January 1, 2012, to January 31, 2015. After implementing the split-sample approach, there were 1,161,732 Veterans classified as the population at risk for model development, of which 940,416 met the aforementioned eligibility requirements for analysis. Likewise, for external validation, 1,977,147 Veterans were classified as the population at risk, with 1,427,473 Veterans meeting the eligibility criteria. Of the 940,416 Veterans available for analysis in the model development dataset, 1,936 experienced a psychiatric hospitalization during the 90-day follow-up. In the external validation dataset, 1,793 Veterans experienced the outcome of interest. Study attrition is presented in Table 1.

**Table 1:** Attrition Table for Model Development and Model Validation.

	Inclusion/Exclusion Criteria	Population

1	All Veterans 65+ years of age during the study period (January 1, 2012, to January 31, 2015)	8,050,371
2	Study population randomly split in half	4,025,184 (Modeling) 4,025,187 (Validation)
3	Veterans with two or more mental-health related diagnoses or at least one mental-health related drug fill during the study period	1,161,732 (Modeling) 1,977,147 (Validation)
4	Veterans with a sufficient eligibility window to support a randomly selected index date in between 365 days of baseline and 90 days of follow-up	940,416 (Modeling) 1,427,473 (Validation)
5	Veterans that experienced a psychiatric hospitalization during follow-up	1,936 (Modeling) 1,793 (Validation)

### Model Development and Validation

**Figure 1:**
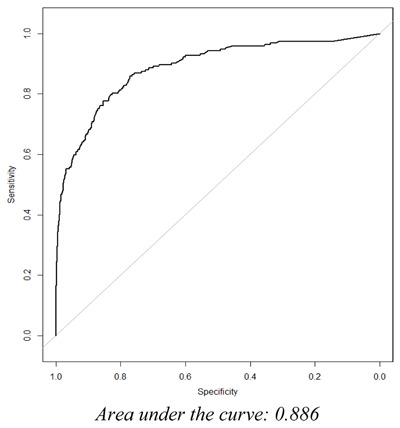
The ROC Curve for the Final Fitted Logistic Model.

**Figure 2:**
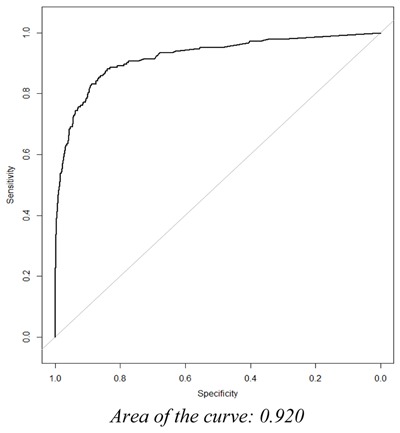
The ROC Curve of the Final Fitted Model during External Validation.

We identified 61 potential candidate predictors during our literature review and consultation with clinical experts specializing in geriatric and psychiatric care. Candidate predictors, representing demographic or clinical concepts, were required to be present in greater than 1 percent of the model development population, as predictor strength is not only a function of the association of the predictor with the outcome, but also takes into consideration the distribution and prevalence of the predictor in the population. Thus, 13 of the 61 potential candidate predictors were removed prior to modeling as they did not meet this criterion. The LASSO regularized regression technique produced a final model containing 9 variables. Utilizing the lambda with the greatest amount of shrinkage whose CVMSE was within 0.5 standard deviation (SE) from the minimum, the final model’s predictive accuracy is represented by a C-statistic of 0.886. See Figure 1 for the receiving operator characteristic (ROC) curve that represents the performance of our fitted logistic model. The model’s predictive accuracy during external validation is represented by a C-statistic of 0.920. See Figure 2 for the receiving operator characteristic (ROC) curve that represents the predictive performance of our final fitted model when applied to the external validation population.

### Model Retained Predictor Variables

The variables within the final fitted model generated are presented in Table 2. Having a previous psychiatric hospitalization in the last year, psychosis, and bipolar disorder were relatively strong predictors of a geriatric psychiatric hospitalization in the next 90 days. Emergency department utilization, number of unique mental-health related diagnoses, and dementia all contributed moderately to the final fitted model. Notable candidate predictors that surprisingly were dropped from the final fitted model included: marital status, service-connectedness, and means test results.

**Table 2:** Fitted Model Generated.

Predictors	Standardized Coefficients

Intercept	–6.865
Number of emergency department visits in the last three months	0.221
Number of unique mental health-related diagnoses	0.110
CAN Score: Probability of hospitalization in the next 90 days greater than 90%	0.009
Previous psychiatric hospitalization in the last year	2.180
Diagnosed with psychosis	0.415
Diagnosed with bipolar disorder	0.656
Number of Beers qualified medication fills	0.005
Diagnosed with dementia	0.296
Attempted suicide and intentional self-inflicted injury	0.002

## Discussion

We have developed and validated a statistical model that predicts a geriatric psychiatric hospital admission in the next 90 days among aging Veterans with an existing mental health condition. We were successful in establishing a high level of predictive accuracy during training (C-statistic 0.886) and validation (C-statistic 0.920) of the prediction model. This predictive accuracy suggests our statistical model is capable of quantifying risk with sufficient performance and can be utilized in identifying Veterans that could benefit from additional care management in order to reduce their risk of psychiatric institutionalization.

Our statistical model suggests that a previous psychiatric hospitalization during baseline is the strongest predictor of a geriatric psychiatric hospitalization in the next 90 days among all the variables considered. Due to the strength of the standardized coefficient for this variable, we also calculated the predictive accuracy of a model that only included this variable. The calculated C-statistic for this variable alone was 0.680. Thus, we can conclude that a previous psychiatric hospitalization is clearly a strong predictor of a future psychiatric admission, but alone is not sufficient in achieving acceptable predictive accuracy. This finding is supported by the literature, which reports that roughly 14 percent of patients that have experienced a psychiatric hospitalization are readmitted within one month after discharge [13,14]. The post-hospitalization period is well recognized as a window of increased risk for mental health-related outcomes, such as suicide and substance abuse relapse, further emphasizing the importance of coordinated outpatient follow-up care, post-discharge [15].

Bipolar disorder and psychosis diagnoses were also strong predictors that contributed substantially to model fit. Previous literature has reported a relationship between these mental health disorders and psychiatric service utilization. For example, a retrospective cohort study, comprised of 2,963 Veterans diagnosed with bipolar disorder, reported that 20 percent (N = 598) of the Veterans were hospitalized psychiatrically during the study’s one-year observation period [16]. Additionally, a study comparing service utilization and associated health care costs between patients with depression and those with bipolar disorder, reported that bipolar patients had greater mean psychiatric inpatient days and emergency room visits in comparison to patients with depression [17]. Patients with bipolar disorder and general psychosis are often institutionalized due to the severe and recurrent episodes of mental health instability associated with these diseases. A recent study reported that medication nonadherence among patients with psychosis was found to increase the risk of future hospitalization substantially (adjusted relative risk = 7.19, p < 0.001), further stressing the importance of treatment adherence and close patient monitoring.

Providers and PACT care managers can utilize prepared reports or perform electronic chart review to identify aging Veterans that may be in need of closer patient monitoring. However, prepared reports can take weeks to produce and electronic chart review is highly inefficient. Identifying who is at risk of psychiatric crisis is often the biggest obstacle to overcome prior to implementing personalized care plan alterations designed to prevent psychiatric hospitalization. Applying this prediction model in a near real-time setting could potentially reduce the incidence of psychiatric institutionalization among older Veterans with mental health disease by assisting clinical teams with their risk assessment workload. In fact, we have begun integrating this validated prediction model into a dashboard reporting system for select VA primary care teams located throughout the country. This dashboard reporting system will use near real-time data to produce a 90-day risk of psychiatric hospitalization for all Veterans, 65 years of age or older with a history of mental health disease, currently assigned to one of the primary care providers found within our list of end users. The VA CDW national production server will serve as the source of underlying data for this dashboard reporting system. Data will be displayed in the dashboard within a secured intranet environment. SQL Server Integration Services (SSIS) packages have been developed to handle the repeated daily data extraction process. SQL Server Reporting Services (SSRS) is being used to produce report visuals and in adding important interactive functions to this dashboard system. The dashboard reporting system will display the average predictive probability for each provider’s patient panel as a whole (see Screenshot 1). Drill-down functionality will allow the user to view individual patient predictive probabilities (see Screenshot 2). Demographic characteristics and details of the predictors identified during model development will also viewable in both reporting views. In addition, the dashboard will also display other meaningful data. For example, the dashboard end user will be able to determine whether a patient at risk for psychiatric hospitalization has been recently referred to mental health services. Furthermore, end users will also be able to use the dashboard to determine if the patient is up-to-date on all nationally recognized mental health-related screening assessments. In short, we are designing a dashboard reporting system that clinical care teams can use to improve care coordination or assist in modifying a patient’s care plan with the goal of preventing future psychiatric admissions.

**Screenshot 1:**
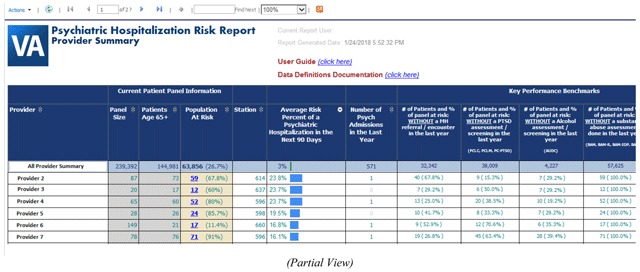
Provider Summary Data View.

**Screenshot 2:**
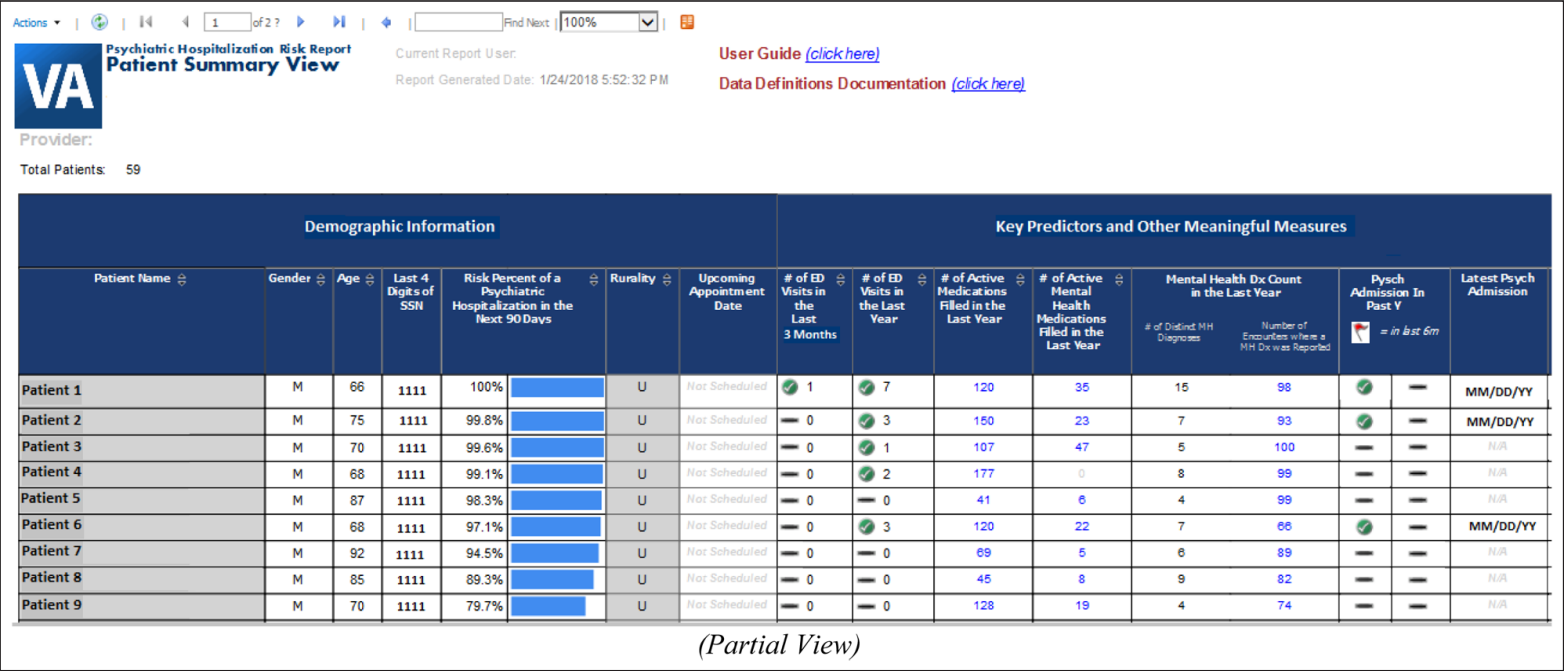
Drill-down Patient Summary Data View.

### Strengths and Limitations

The main strength of this study is that the prediction model is trained and validated on over two million Veterans that were receiving care from multiple VA facilities located throughout the country. This increases the generalizability of the model and its usefulness across the VHA. To further assist with generalizability, we also used a modern regression technique during model development that is designed to specifically analyse high-dimensional data without overfitting. Unfortunately, dated techniques such as stepwise selection and ordinary least squares regression are still common approaches utilized for predictor selection, even though it is well known that these approaches are prone to producing overinflated regression coefficients and R-squared estimates [18]. An additional strength of this study is that substantial effort was made to correctly identify both VA & Non-VA psychiatric hospitalizations for the outcome, which further improves the completeness of our analysis as many VAMCs do not always have a sufficient number of beds for geriatric patients in need of inpatient psychiatric care. Including only VA psychiatric hospitalizations would underrepresent outcome occurrence among the target population.

The main limitation of this study was that we relied upon ICD and CPT codes as a means to identify several of the candidate predictors. The literature does suggest that the accuracy of diagnostic coding has increased in the last decade, due to the rising trend in research relying upon claims data [19]. However, VA institutions are federally funded and place less emphasis on insurance providers and billing, which further impacts the reliability of these coding systems [20]. As a result, there is likely some degree of underreporting influencing the prevalence of the clinical condition and laboratory predictors analysed. In addition, even though the validated prediction model is highly generalizable for older Veterans located throughout the country, it may not perform well among other populations outside the VA. Additional external validation on an outside population is warranted prior to outside entities relying on this model in their endeavour to improve patient outcomes.

## Conclusion

This predictive model is capable of quantifying the risk of a geriatric psychiatric hospitalization with acceptable performance. The ability to predict the risk of a geriatric psychiatric hospitalization allows for the development and implementation of patient care interventions that could potentially prevent psychiatric crisis, especially among older Veterans with a history of mental health disease. Due to the accuracy of the validated prediction model we are able to enhance the decisions made by VA primary care teams in identifying aging Veterans at risk for psychiatric hospitalization by way of an interactive dashboard reporting system. Further investigation is warranted in determining the utility of this validated model for populations outside the VA.

## Additional File

The additional file for this article can be found as follows:

10.5334/egems.207.s1 Appendix A Supplemental Tables.

**Appendix A**Supplemental Tables. https://doi.org/10.5334/egems.207.s1
