# Supplementary material for: Predicting Psychiatric Hospitalizations among Elderly Veterans with a History of Mental Health Disease
Source: EGEMS (Wash DC). 2018 May 17;6(1):7. doi: 10.5334/egems.207 (PMC5982950; doi:10.5334/egems.207)
Supplement: Appendix A — Supplemental Tables. [file egems-6-1-207-s1.pdf]

Appendix A: Supplemental Tables

Table A1. Single-Level HCUP CCS Categories Representing a Mental Health-Related Diagnosis

Single-Level CCS Diagnosis Categories	CCS Category Description
650	Adjustment disorders
651	Anxiety disorders
652	Attention-deficit, conduct and disruptive behavior disorders
653	Delirium, dementia, and amnestic and other cognitive disorders
656	Impulse control disorders
657	Mood disorders
658	Personality disorders
659	Schizophrenia and other psychotic disorders
660	Alcohol-related disorders
661	Substance-related disorders
662	Suicide and intentional self-inflicted injury
663	Screening and history of mental health and substance abuse codes
670	Miscellaneous mental health disorders

Table A2. VA Drug Class Codes Representing a Mental Health-Related Drug Fill

VA Drug Class	Drug Classification
AD100	Alcohol Deterrents
AD400	Antidotes, Deterrents, and Poison Control Exchange Resins
AD900	Antidotes/Deterrents, Other
CN300	Sedatives/Hypnotics
CN301	Barbituric Acid Derivative Sedatives/Hypnotics
CN309	Sedatives/Hypnotics, Other
CN400	Anticonvulsants
CN500	Antiparkinson Agents
CN600	Antidepressants
CN601	Tricyclic Antidepressants
CN602	Monamine Oxidase Inhibitor Antidepressants
CN609	Antidepressants, Other
CN700	Antipsychotics
CN701	Phenothiazine/Related Antipsychotics
CN709	Antipsychotics, Other
CN750	Lithium Salts
CN800	CNS Stimulants
CN801	Amphetamines
CN802	Amphetamine Like Stimulants

Table A3. VHA Stop Codes Representing Outpatient Visits

Primary Stop Code	Secondary Stop Code	Visit Description
131	-	Urgent Care Visit
130	-	Emergency Room Visit
323, 348	-	Primary Care Visit
156-157, 170-178	-	Home-Based Primary Care Visit
118, 121	-	Home Health Visit
502, 509-510, 512, 550, 557-558, 576–577	125	Mental Health Social Work Visit
125	-	Non-Mental Health Social Work Visit
Any Stop Code not equal to 502, 509-510, 512, 550, 557-558, 576-577	125	Non-Mental Health Social Work Visit
